# Influence of variable hinge positioning on coronal and sagittal alignment in uniplanar medial open‐wedge high tibial osteotomy

**DOI:** 10.1002/jeo2.12049

**Published:** 2024-06-17

**Authors:** Jannik Frings, Alexander Korthaus, Philip Linke, Tim Rolvien, Julian Stürznickel, André Strahl, Matthias Krause, Karl‐Heinz Frosch

**Affiliations:** ^1^ Department of Trauma and Orthopaedic Surgery University Medical Center Hamburg‐Eppendorf Hamburg Germany; ^2^ Department of Osteology and Biomechanics University Medical Center Hamburg‐Eppendorf Hamburg Germany; ^3^ Department of Trauma Surgery, Orthopaedics and Sports Traumatology BG Klinikum Hamburg Hamburg Germany

**Keywords:** coronal and sagittal alignment, hinge, HTO, medial open‐wedge high tibial osteotomy

## Abstract

**Purpose:**

There is a high risk of increasing the posterior tibial slope (PTS) during medially opening‐wedge high tibial osteotomy (mowHTO). Most recently, the idea of intentional simultaneous coronal and sagittal correction, using uniplanar cutting techniques has emerged. This study aims to examine the influences of variable hinge positioning and osteotomy gap height on the sagittal and coronal plane.

**Methods:**

Twenty uniplanar mowHTOs were performed in standardised (left) solid‐foam proximal tibia models. In the different models, hinge position was rotated stepwise by 10°, between 90° (cutting straight medial to lateral) and 0° (cutting straight anterior to posterior) (*n* = 10). Additionally, gap heights of 5 and 10 mm were produced and analysed. Logistic regression analysis was performed to calculate a predictive regression formula for the relation between gap height, hinge rotation and the changes of medial proximal tibia angle (MPTA), medial and lateral PTS.

**Results:**

Between cutting angles of 90 and 20°, the MPTA was mainly influenced by the gap height (0.95° MPTA per 1 mm gap height), but not by the cutting angle. Between 20 and 0°, the MPTA was decreased by 3.6° per 10° of rotation, but not by the gap height. Between cutting angles of 90 and 10°, the PTS was increased linearly by 0.97° per 10° of rotation and by 0.61° per 1 mm gap height.

**Conclusion:**

In mowHTO with lateral hinge positions, the MPTA is mainly influenced by gap height. Changes of PTS can be avoided by a straight lateral hinge position. In posterior hinge locations (0‐20°), changes of MPTA are mainly caused by hinge rotation, but not by gap height.

**Level of Evidence:**

Level III, Case–control study.

**Abbreviations**
HTO: high tibial osteotomy
latPTS: lateral posterior tibial slope
medPTS: medial posterior tibial slope
mowHTO: medial open‐wedge high tibial osteotomy
MPTA: medial proximal tibia angle
OA: osteoarthritis
PTS: posterior tibial slope

## INTRODUCTION

Coronal and sagittal malignment of the lower extremity causes an uneven pressure distribution on the articular cartilage of the knee, which eventually leads to the development or progression of unicompartmental osteoarthritis [3, 21]. This is further increased by ligamentous instability, such as anterior cruciate ligament (ACL) deficiency [1, 7, 14]. At the same time, lower limb malalignment alters intraarticular kinematics, increasing the tension forces on the native or reconstructed ACL [11, 13, 25]. In a cadaveric study, a linear increase of ACL graft tension was found, in presence of varus malalignment in combination with secondary lateral ligament deficiency (varus thrust) [25]. In these cases, medial open‐wedge high tibial osteotomy (mowHTO) has been reported to yield good clinical results and can be combined with simultaneous ACL‐R [5, 6, 9, 14]. Nevertheless, mowHTO bears a high risk of unintentional alteration of the sagittal plane, depending on the angulation of the osteotomy and the subsequent rotation of the contralateral hinge [15, 18]. A posterolateral rotation of the hinge of 4.9° will increase the posterior tibial slope (PTS) by 3.3° [15, 18]. In turn, an increasing PTS of 5° rises the tensile load on the ACL by 75% [12, 25]. Eventually, a PTS of >12° is associated with a significantly higher graft failure rate of 59% and is, therefore, considered to be the upper limit [2, 18, 24].

Against this background, increased attention has been brought to the influence of the hinge position on the sagittal alignment, in the course of uniplanar HTO. A recent simulation study has proposed an anterolateral hinge placement for a PTS‐neutral correction during mowHTO [8]. In another study, successful simultaneous slope reduction during mowHTO was achieved by a medial soft‐tissue release, which was combined with a release of ‘the posterolateral aspect, close to the lateral hinge’, thus creating an intentional asymmetric hinge [26]. However, to the best of our knowledge, no study has examined the influence of various cutting directions and subsequent continuous hinge rotation on both planes, in a standardied set‐up.

The goal of this study was, therefore, to test the influence of uniplanar HTO on the coronal and sagittal plane using various hinge positions. We hypothesised that the PTS can be addressed simultaneously in uniplanar mowHTO, by intentional hinge rotation and with regard to the opening height of the osteotomy gap. It was further hypothesised that the changes of either plane were predictable, depending on the applied hinge angulation and gap size, allowing for an individualised use of mowHTO.

## MATERIALS AND METHODS

### Experimental set‐up

For this study, a series of uniplanar HTOs was performed on standardised solid foam models of left proximal tibias (4th SawBone®). In each model, one osteotomy was performed. In doing so, the hinge positions were determined by variable angulations of the osteotomies in relation to an orthogonal line to the posterior tibial condyles (PTC). In order to create reliable and reproducible results, all osteotomies were performed in the same monoplanar fashion.

**Figure 1:**
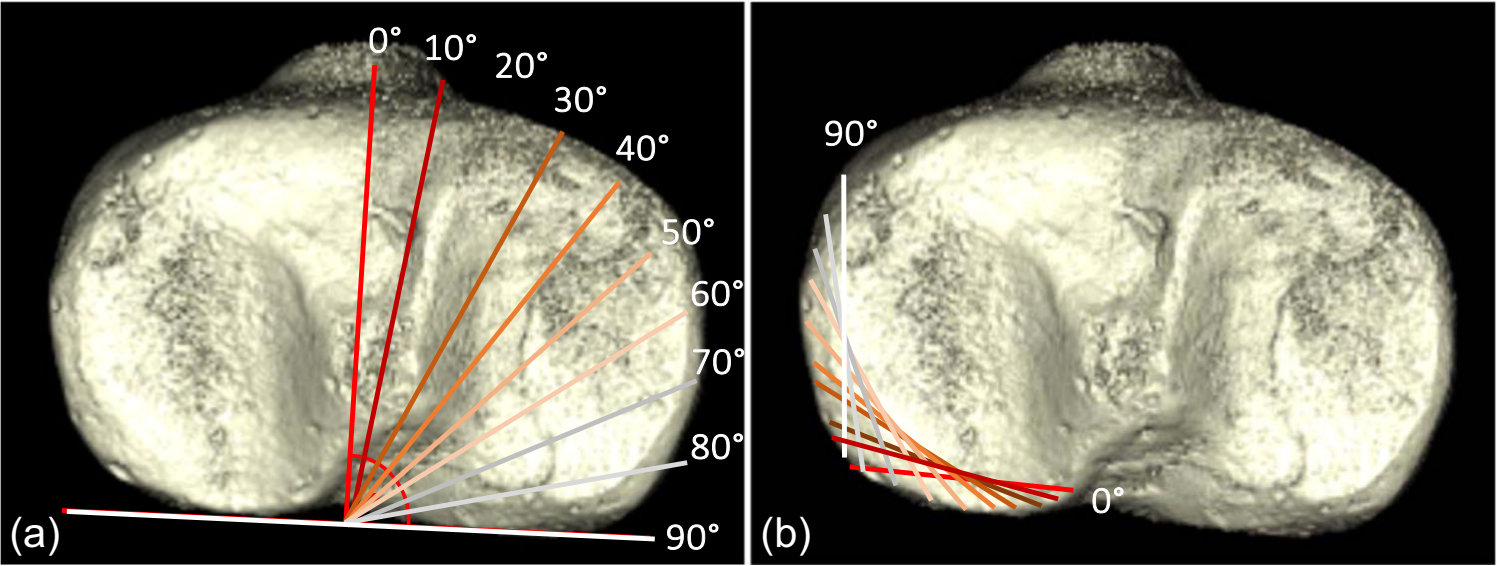
Schematic of the planned stepwise posterior hinge rotation. The cutting angulation was increased stepwise by 10° from a straight anterior‐to‐posterior cut (0°) to a medial‐to‐lateral cut (90°) (a). Accordingly, the contralateral hinges rotated from a straight lateral hinge (90°) to a strictly parallel posterior hinge (0°) (b).

The angulations were performed in steps of 10°, starting at 0° with a straight anteroposterior cut, until a 90° angle in terms of an ideal mowHTO was reached (*n* = 10) (Figure 1). The osteotomies were performed in a total of two rounds, so each osteotomy was combined with an opening of 5 and 10 mm (*n* = 2 × 10 = 20), with consideration of the technical feasibility.

### Osteotomy

**Figure 2:**
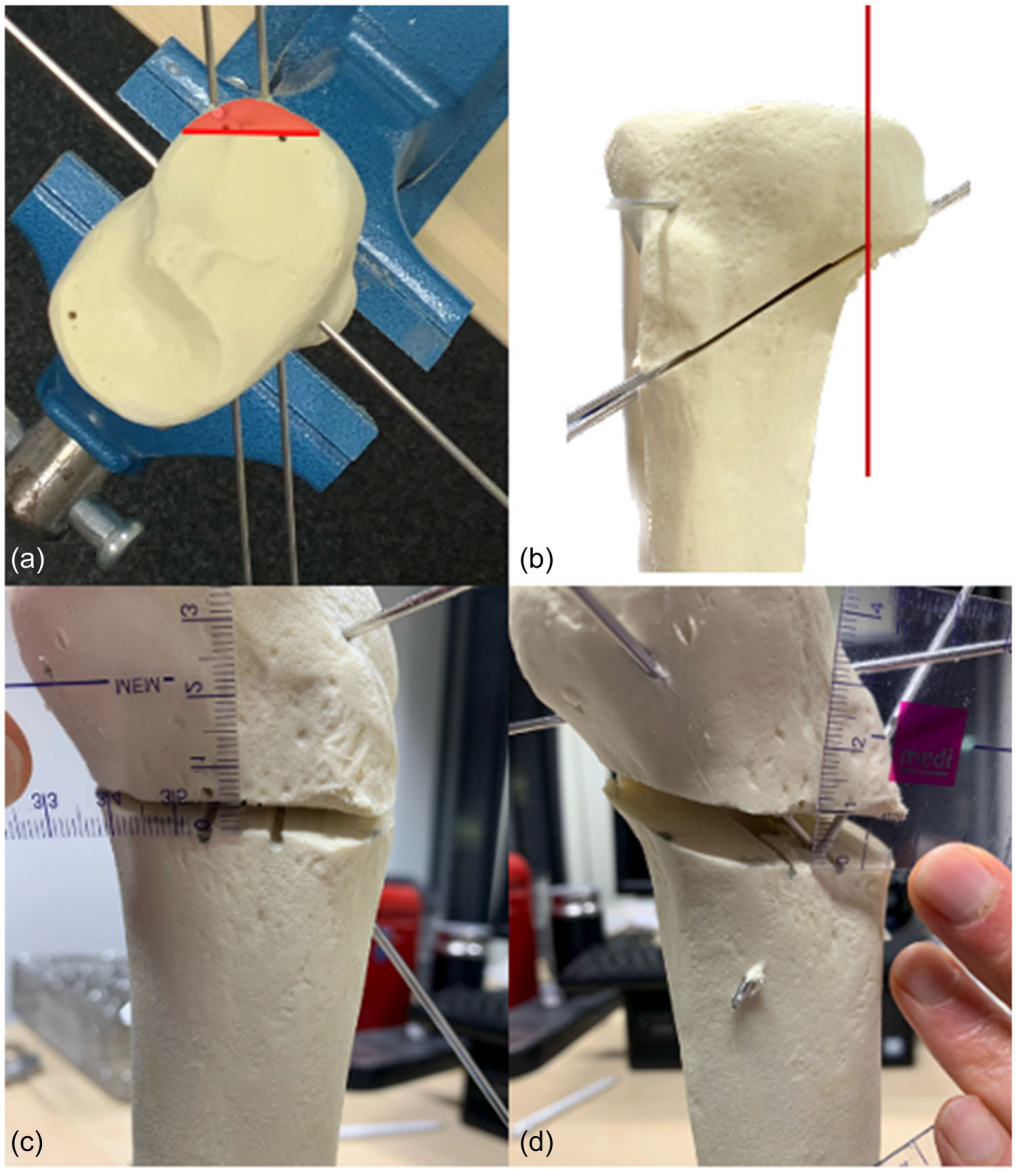
Experimental osteotomy set‐up. A straight anterior‐to‐posterior reference pin was placed orthogonally to the posterior tibial condylar line. Two guide wires were inserted in the desired cutting angle, to mark the direction of the osteotomy. The hinge was marked with an angle of 90° to the guide pins and with a standardised thickness of 5 mm from the contralateral cortex (a). Two additional *k*‐wires were used to protect the planned hinge. Hinge symmetry was thoroughly ensured during the osteotomy (b). After the insertion of a hinge protection wire, the osteotomy gaps were opened 5 (c) or 10 mm (d). The spreader was inserted exactly between the two guide wires, to ensure reproducible and exact results.

The models were fixed in a screw clamp, ensuring a standardised orientation within the clamp. A central guide wire was placed proximal to the tibial tubercle, which indicated the exact orthogonal direction (90°) in relation to the PTC. The planned cutting direction was defined by a guide pin (2.0 mm *k*‐wire), which was introduced through an aiming device (AAP Implants) in the planned cutting direction. The height of the entrance point was defined to be 16 cm from the distal end of the models, which presented a uniform and reliable landmark in the tibia models used. The height of the hinge was defined to be at 50% of the height of the lateral aspect of the tibial head. A second parallel *k*‐wire was added, in order to allow for a precise cutting plane during the osteotomies. Accordingly, the hinge point was located approximately 1 cm below the joint line. The guide pins were then used for hinge orientation and thickness. The hinge was marked in 90° to the guide pins and with a standardised distance of 5 mm to the contralateral cortex (Figure 2a). Three parallel *k*‐wires (2.0 mm), which were drilled through the hinge line, served as a mechanical border for the saw blade during the osteotomy (Figure 2b). All osteotomies were performed using an oscillating saw (TRS Modular Drive, DePuy Synthes). An additional *k*‐wire (2.0 mm) was drilled though the intact hinge, to prevent hinge fracture. After the sawing cut, a scaled spreader was introduced at the exact position of the guide pin. This position was chosen to achieve the best possible reproducibility, as hinge rotation was performed within a full range of 90°. The osteotomy gaps were then opened to 5 mm (group5) and 10 mm (group10), with consideration of a 1 mm cutting defect. The gap heights were measured and verified repeatedly, at the position of the spreader, using a medical ruler (Figure 2b,c). Two crossing *k*‐wires (2.0 mm) were used to fix the osteotomy, close to the medial cortex. After the spreader was retracted, the correct height of the gap opening was reconfirmed.

### Radiological examination and measurement

In order to allow for a precise and multiplanar measurement, radiological examination was performed using a Cone Beam CT (SCS MedSeries® H22, SCS). The models were placed in supine position on a flat carbon tray, while a standardised position on the tray was reinforced prior to the examination.

Radiological analysis was performed using an open‐source medical image viewer (Horos, version 3.3.6; The Horos Project). The height of the osteotomy gaps was measured on sagittal and coronal planes, in order to verify the previous measurements. Coronal alignment was defined by the medial proximal tibia angle (MPTA), while the medial and lateral PTS were measured to define sagittal alignment. The MPTA was measured on an image in the coronal plane, which was referenced to the most proximal part of the tibial eminentia in the sagittal plane. It was defined to be the angle between the proximal articular surface and the vertical reference line of the image viewer. Due to the standardised position of all models during the CT scan, the latter was considered to be parallel to the tibial crest. The medial (medPTS) and the lateral (latPTS) PTS were measured in sagittal images of the medial and lateral tibial joint surfaces. For this purpose, the most central sagittal images of the concerning joint surfaces were chosen. The PTS was defined to be the angle between the anterior tibial cortex and the medial or lateral joint surface, respectively [16].

All measurements were referenced to the values measured in the native SawBone® model.

### Sample size calculation

The primary statistical analysis based on a multiple regression model with two independent and one dependent variable. So far only few case series are available to estimate the statistical power. Hence, under the conventional assumption, this study expected a large experimental effect of *f* ^2^ = 0.57. This represents a squared multiple correlation of *R*² = 0.375. With a *⍺* risk of 0.05, a statistical power of 0.8 and an effect size of *f*² = 0.57 a total sample size of 20 samples is required to calculate a multiple linear regression with two independent predictors. This number of cases is adequate to achieve sufficient statistical power. An effect size of *f*² = 0.57 represents a large effect to detect significant beta‐weights in the regression analysis (G*Power software ver. 3.1.9.2; Heinrich‐Heine‐Universität Düsseldorf).

### Statistical analysis

Data compilation and graph preparation were performed in Excel, version 16.66.1 (Microsoft). Statistical calculations were performed in SPSS Statistics, version 290 (IBM). Multiple linear regression analyses were conducted to investigate the influences of gap opening and hinge angulation on MPTA, medPTS and latPTS. The latter were considered the dependent variables, while gap size and hinge angulation were regarded as independent variables. With respect to the graphical analyses, additional linear regression models were conducted, in order to investigate sectional correlations.

Data were presented in mean and standard deviations, as well as ranges of all values. The statistical level of significance was set to *p* < 0.05.

## RESULTS

The native SawBone® model presented an MPTA of 85.2°, latPTS of 11.5° and medPTS of 10.5°. A total of 20 open‐wedge osteotomies were performed, according to the previously planned protocol. Among all osteotomies, no hinge fractures occurred.

In a hinge angle of 0° (straight anterior to posterior cut), MPTA was 82.9° (group5) and 83.7° (group10). In a hinge angle of 90° (straight medial to lateral cut), the MPTA was 88.37° (group5) and 92.77° (group10).

**Figure 3:**
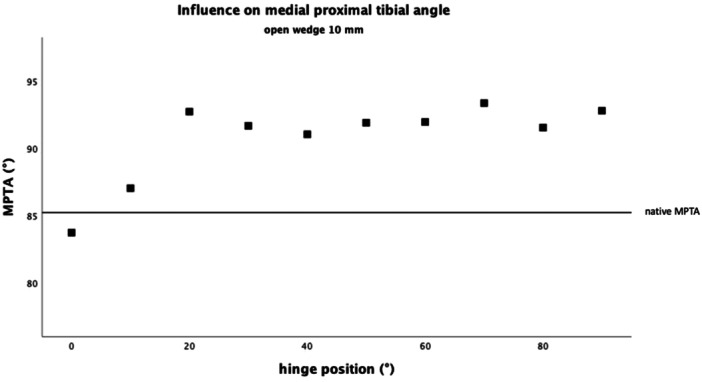
Influence on the medial proximal tibial angle (MPTA) with a gap height of 5 mm. There was no linear correlation between the hinge position and the MPTA. However, depending on the hinge position, the influence of the opening height varied.

**Figure 4:**
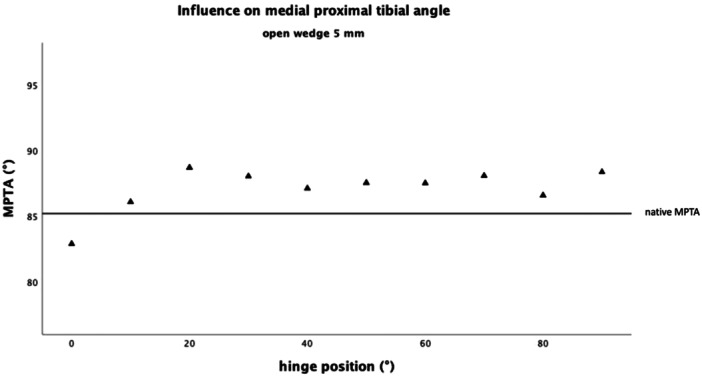
Influence on the medial proximal tibial angle (MPTA) with a gap height of 10 mm. There was no linear correlation between the hinge position and the MPTA. However, depending on the hinge position, the influence of the opening height varied.

No general linear relation was found between the hinge rotation and the MPTA (Figures 3 and 4). In particular, between 20° and 90°, stepwise posterior hinge rotation of 10° had no significant influence on the MPTA, while gap height increased the MPTA by 0.95° (*F*_(2, 13)_ = 60.84, *p* < 0.001, *n* = 15) per 1 mm (Figures 3 and 4). However, between the hinge positions of 0–20°, stepwise posterior hinge rotation of 10° decreased the MPTA by 3.6° with every 10° of hinge rotation, while the gap height had no significant influence (*F*_(2, 3)_ = 19,26 *p* = 0.019, *n* = 5).

**Figure 5:**
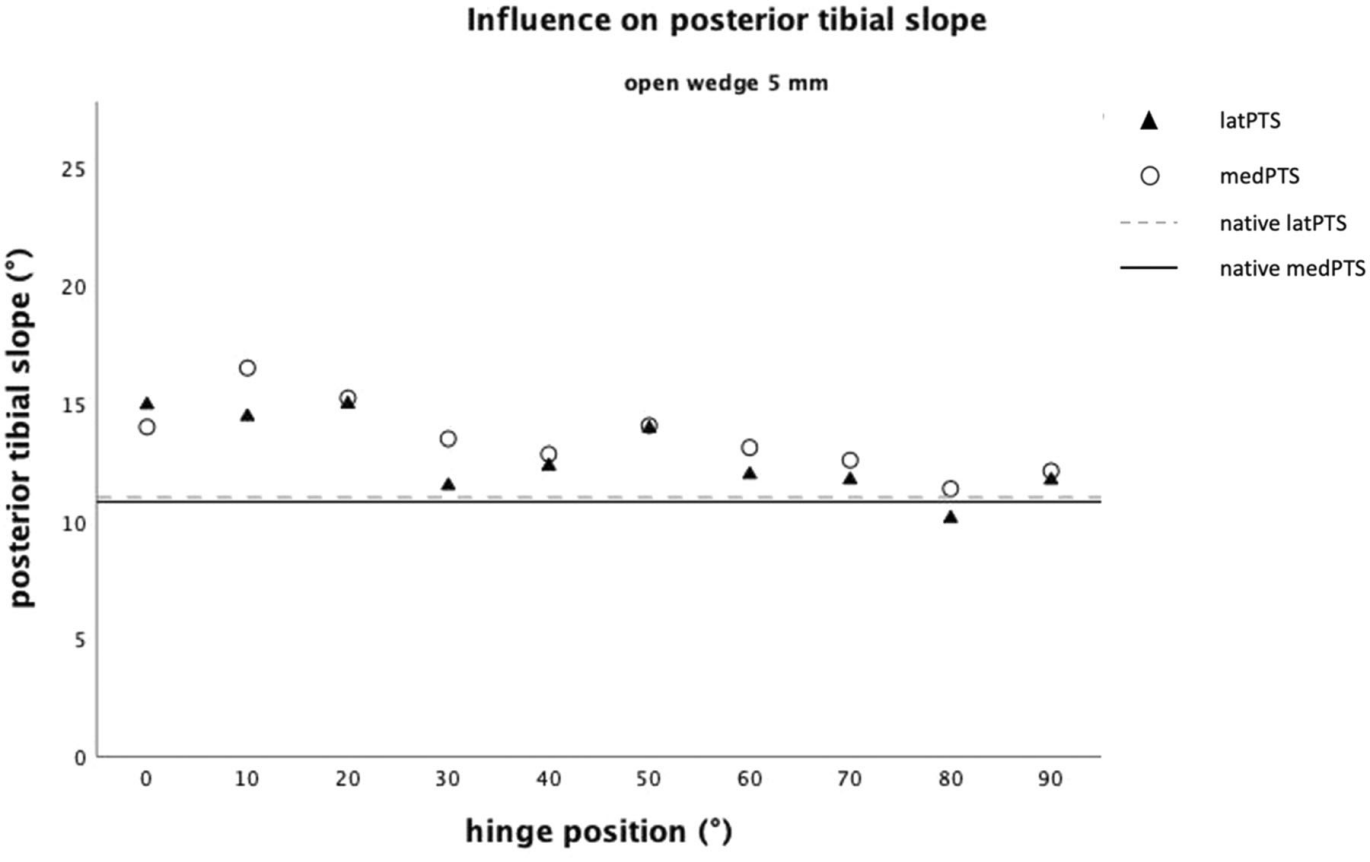
Influence on the sagittal plane, defined by the medial (medPTS) and lateral (latPTS) posterior tibial slope (PTS) with a gap height of 5 mm.

**Figure 6:**
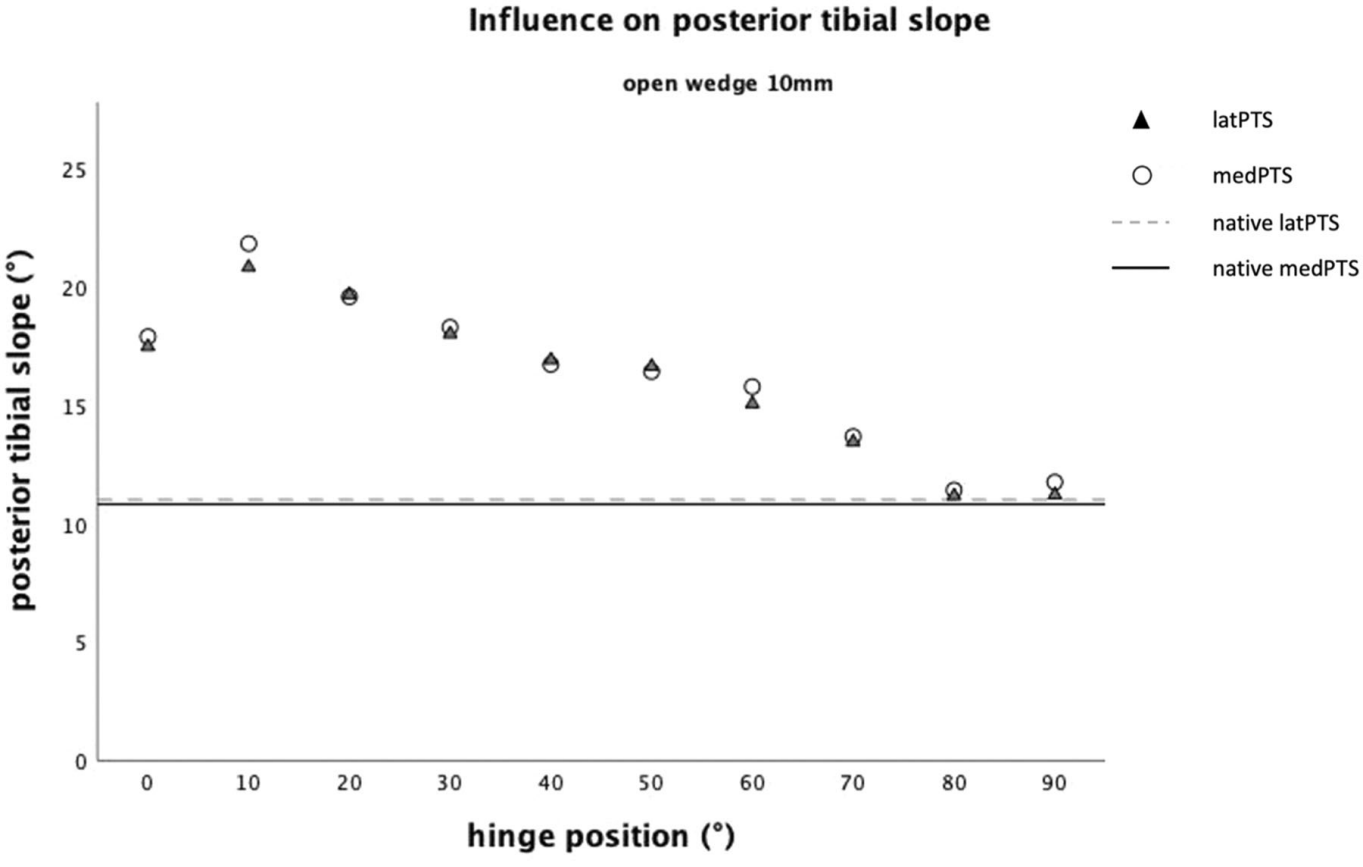
Influence on the sagittal plane, defined by the medial (medPTS) and lateral (latPTS) posterior tibial slope (PTS) with a gap height of 10 mm.

In the sagittal plane, linear relations between both hinge rotation and gap height and the medial and lateral PTS were observed. The medPTS was increased by 0.68° with stepwise posterior hinge rotation of 10°, while 1 mm of additional gap height increased the medPTS by 0.72° (*F*_(2, 17)_ = 33.25, *p* = 0.001, *n* = 19). The latPTS was increased by 0.67° with stepwise posterior hinge rotation of 10°, while an additional gap height of 1 mm increased the latPTS by 0.63° (*F*_(2, 17)_ = 29.24, *p* < 0.001, *n* = 19) (Figures 5 and 6).

Since the influence of the gap height on the PTS increased with progressing posterior hinge rotation, the influence of hinge rotation on PTS was additionally analysed separately, for group5 (Figure 5) and group10 (Figure 6). In group5, posterior hinge rotation of 10° increased the medPTS by 0.40° (*F*_(2, 7)_ = 5.78, *p* = 0.033, *n* = 9) and the latPTS by 0.34° (*F*_(2, 7)_ = 11.82, *p* = 0.006, *n* = 9). In group10, the influence of posterior hinge rotation increased. Accordingly, stepwise posterior rotation of 10° increased the medPTS by 0.96° (*F*_(2, 7)_ = 19.16, *p* < 0.001, *n* = 9) and the latPTS by 0.97° (*F*_(2, 7)_ = 18.75, *p* = 0.002, *n* = 9). Based on the above findings, latPTS and medPTS were used to calculate the mean parameter ‘PTS’. Accordingly, PTS was increased by 0.97° by posterior hinge rotation of 10° and by 0.61° with increasing gap height of 1 mm (*F*_(2, 7)_ = 19.39, *p* = 0.001, *n* = 9). Oddly, an initial increase of PTS was observed between 0° and 10° for the PTS (Figure 6).

Against the background of the above findings and based on the linear regression analysis, a multiple linear regression formula was postulated, with the aim to predict the changes of medPTS and latPTS, influenced by stepwise posterior hinge rotation and gap height in mowHTO.

The derived formula was: PTS = 19.39 + 0.97 × *hinge rotation* + 0.61 × *gap height*.

## DISCUSSION

The most important finding of this study was that the simultaneous correction of the sagittal and coronal planes was achieved in uniplanar mowHTO, depending on variable hinge rotation and gap height. The MPTA was mainly influenced by the height of the osteotomy gap, while the influence of rotating hinge predominated in more posterior hinge positions. The PTS was influenced by both, hinge rotation and gap height, showing an increasing influence of the gap height with progressive posterior hinge rotation. Based on the findings, a predictive formula was developed to anticipate the influence of mowHTO on PTS.

In the recent literature, there has been an increasing awareness for the relation between the sagittal and the coronal plan changes in mowHTO, mainly to avoid an incidental influence of the PTS. Particularly, in presence of concomitant ligamentous instabilities, as in ACL deficient knees, incidental changes in the sagittal plane become particularly important. An increase of the PTS increases the ACL strain and favours subsequent graft failure [12, 24].

In this regard, Nha et al. performed a meta‐analysis to examine the general influence of opening or closing wedge HTO on the sagittal plane. They observed an average increase of PTS by 2.02° after opening wedge HTO, while closed‐wedge HTO was associated with a decreasing PTS [18]. These observations may be explained by the unique triangular shape of the proximal tibia, causing an asymmetrical opening or closure in HTO [10]. Further studies have identified specific technical factors, which contribute to these above‐mentioned PTS changes. These may comprise inadequate and asymmetric bone resection in closing‐wedge HTO, incomplete osteotomy of the posterolateral aspect of the tibial plateau in opening‐wedge HTO or accidently posterolateral hinge rotation due misguidance of the guide pins [4, 15, 22]. Furthermore, anterior plate positioning may be a cause for an increasing PTS in mowHTO [20]. In a three‐dimensional computer tomography (CT) controlled case series of 19 mowHTO, Moon et al. have found an incidental posterolateral hinge rotation of 4.9°. Although not clinically visible, this posterior hinge malrotation was associated with a significant increase of the PTS by an average of 3.3° [15]. In a computational simulation model, Eliasberg et al. picked up on these findings and proposed a hinge axis rotation of 102.6° relative to the posterior condylar axis, in order to achieve a PTS‐neutral coronal correction during mowHTO [8]. This observation may be explained by a compensational effect, with regard to the tibial anatomy. On the other hand, the definition of the ‘hinge axis’ is not uniform throughout literature, which could be a source of bias in this context [8]. Yet, hinge rotation was consistently identified to be a controllable influence factor for intentional changes of the PTS [8].

The findings of this study suggested a linear relationship between hinge rotation and PTS. With progressing posterior hinge rotation, a continuously increasing PTS was observed, indicating the importance of a strict lateral hinge position for isolated coronal plane correction. Therefore, from our point of view, the increase in PTS described in the literature could be due to the fact that the hinge position is not strictly lateral. This could be a result of an oblique osteotomy, for example. Since a deviation of the hinge position by as much as 20° leads to a change. Yet, in presence of concomitant posterior cruciate ligament instabilities, this knowledge may be of interest, to allow for simultaneous varus correction and enlargement of PTS. Accordingly, a slightly more posterolateral hinge rotation might be a technical option in presence of a concomitant varus deformity and a flat PTS. At the same time, according to the findings of this study, changes of hinge rotation between 20° and 90° had little influence on the MPTA, which was mainly influenced by the opening height of the osteotomy gap. These findings were also observed by Eliasberg et al. [8]. Based on the results of this study, we proposed a formula (PTS = 19.39 + 0.97 × *hinge rotation* + 0.61 × *gap height*) to predict the aspired changes in the coronal and sagittal plane, with regard to the factors opening height and hinge rotation.

Vice versa, when aiming to correct a varus deformity and a pathologically increased PTS, extreme anterolateral hinge rotation may be a technical option to combine, both varus correction and slope‐reducing osteotomy in a single‐cut procedure [8]. However, these considerations are based on virtual concepts, and so far, lack clinical applicability. In the clinical practice, there is a tendency towards incidental posterolateral hinge rotation, even if a strict lateral position is aspired [15]. Therefore, anterolateral hinge placement would require even more extreme cutting angulations, which, in contrast to simulation studies or sawbone models, is distinctively limited by the (postero‐)medial soft tissue. Against this background, biplanar cutting techniques may be more suitable, as they allow for a reliable and reproducible varus correction and slope reduction [17]. In this study, however, analysis of a continuously rotating hinge position required an uniplanar cutting technique, as anterior‐to‐posterior cutting directions were not suitable for biplanar cutting techniques. This may be a source of bias of this study. In this context, it should also be mentioned that hinge placement in single‐cut concepts is not entirely transferable to real patients. Besides technical considerations like cutting direction and hinge protection, the accessibility of the bone is limited by surrounding soft‐tissue, which may cause an increased risk for neurovascular structures [19]. Another, often underestimated factor when transferring these theoretical considerations into clinical application, is intraoperative precision. In reality, intraoperative measurement is even more limited, compared to the good general accessibility of an artificial bone model. Besides the gap height, this particularly applies to the exact hinge rotation. In this study, hinge rotation was referenced to the posterior tibial condylar line, which has been described before [8]. However, in clinical practice, this referencing method is not feasible, which limits the exact transfer to reality to a certain extent. Even postoperatively, a CT scan is seldomly performed following HTO, so the range of variation may presumably remain underestimated and possibly undetected.

Nevertheless, effective ‘hinge rotation’ is not only a question of precise planning and positioning of guide pins. It is also a product of accurate cutting, reproduction of preoperative planning and hinge symmetry [23]. Moon et al. have outlined the importance of an intact posterior cortex, in order to avoid incidental PTS alteration [15]. Yet, (functional) hinge asymmetry may also result from a pronounced lateral hinge position, which subsequently increases hinge flexibility in the sagittal plane. Weiler et al. have postulated technical considerations for simultaneous varus correcting and slope‐reducing uniplanar HTO, suggesting a pronounced lateral hinge position [26]. In their study, slope reduction was achieved by synchronous anterior compression and posterior insertion of a spreader, taking advantage of the flexible hinge [26]. Although Weiler et al. focussed on isolated slope correction, another technique described by Müller et al., which is used for simultaneous correction in both planes, also shows that a high level of experience and intraoperative precision is required [26]. At the same time, however, functional hinge asymmetry may also occur unnoticed, which eventually results in an unintentional effective hinge rotation [15].

There were some limitations to this study. First, the data was obtained using artificial bone models without the surrounding soft tissue, which does not reflect the exact anatomical reality. Also, the biomechanical properties of artificial bone may differ from those of vital bone tissue, and therefore, imply a different behaviour of the contralateral hinge during the correction. Second, due to the use of isolated tibial bone models, there was no possibility to visualise the changes of the leg axis in the coronal plane. Therefore, all changes were evaluated by observation of the according changes of MPTA and PTS. Against this background, several different methods for measuring PTS have been postulated. However, with regard to different imaging modalities, no method has been shown to be superior. The utilised technique was most feasible for CT‐based measurement. In the clinical application, relevant variabilities, due to individual anatomic variations of the tibial tubercle, must be presumed. However, this limitation was not relevant in this study as each model represented the same tibial anatomy. It should also be mentioned that the use of *k*‐wire to avoid hinge fractures in the bone model may have different effects than in human bone. Third, in this study, hinge placement and rotation were determined by visual measurement, which is usually not possible in the clinical practice. Usually, hinge orientation is confirmed fluoroscopically, only allowing for an approximate estimation of the hinge rotation. This may present another limitation for the transfer of our findings into clinical practice. At the same time, however, this point provided the impetus to discuss whether intraoperative three‐dimensional CT scans and navigated surgery could have a positive influence here.

In conclusion, posterior hinge rotation and gap size both significantly influence the sagittal plane during mowHTO. Between a straight lateral (90°) to posterior hinge position of 20°, the MPTA was mainly influenced by the gap height, with a decreasing influence in posterior hinge locations. The PTS was influenced by both, hinge rotation and gap height, with an increasing influence of the gap height in more posterior hinge locations. In the case of mowHTO, the PTS can be best controlled by a purely lateral hinge position.

## AUTHOR CONTRIBUTIONS

Jannik Frings and Alexander Korthaus drafted and wrote the manuscript. Jannik Frings and Philip Linke performed the osteotomies. Julian Stürznickel and Tim Rolvien performed the radiological analysis. Jannik Frings and Philip Linke carried out the measurements. Jannik Frings and Matthias Krause developed the study design. Matthias Krause and Karl‐Heinz Frosch provided the theoretical background for the conception of the study. Jannik Frings performed the statistical analysis and data interpretation. Jannik Frings designed the figures. Matthias Krause, Karl‐Heinz Frosch and Alexander Korthaus critically reviewed and revised the manuscript. Jannik Frings is the corresponding author. All authors reviewed and approved the final version of the manuscript and take responsibility for its content.

## CONFLICT OF INTEREST STATEMENT

The authors declare no conflict of interest.

## ETHICS STATEMENT

Ethical consultation was not necessary for this study as no human data was used, and all studies were carried out on sawbones.

## Data Availability

The data, materials and code used in this study are available upon reasonable request. For inquiries regarding access, please contact the corresponding author.
